# Gut microbiota induces immune-related alterations in gene expression, RNA methylation, and metabolism in glioblastoma revealed by single-cell and spatial multi-omics

**DOI:** 10.3389/fimmu.2026.1899954

**Published:** 2026-07-15

**Authors:** Mingcong Chen, Xiang Wang, Gang Peng, Lihe Jiang, Hao Liang, Ping Cui

**Affiliations:** 1 School of Public Health, Guangxi Medical University, Nanning, China; 2 Life Sciences Institute, Guangxi Medical University, Nanning, China; 3 Department of Transplantation, The Second Affiliated Hospital of Guangxi Medical University, Nanning, China; 4 Department of Gastroenterology, The People’s Hospital of Longmatan District, Luzhou, China; 5 School of Basic Medical Sciences, Youjiang Medical University for Nationalities, Baise, China

**Keywords:** ABX, Epha, glioblastoma (GBM), gut-brain axis, m6A methylation

## Abstract

Glioblastoma (GBM) is a highly malignant tumor with poor prognosis and limited effective treatment options. Emerging studies have suggested that gut microbiota may influence glioma progression through the gut-brain axis, though the precise mechanisms remain largely unclear. In this study, we employed a comprehensive multi-omics approach—encompassing single-cell transcriptomics, spatial transcriptomics, metagenomics, metabolomics, and m6A-seq—to investigate how antibiotic-induced gut microbiota disruption impacts glioma progression in a mouse model. Gene expression analysis revealed significant alterations in antibiotics-treated mice (ABX-treated mice), including reduced expression of Epha6 and upregulated expression of Tead1, key genes associated with glioma progression and immune modulation. Spatial transcriptomics and metabolomic profiling identified reduced methionine levels in gliomas of ABX-treated mice, linking gut-derived metabolite changes to epigenetic regulation via m6A methylation. Single-cell RNA sequencing further demonstrated an increased proportion of AC-like cells, disrupted intercellular communication, and aberrations in the EPHA and NRXN signaling pathways. These findings highlight the interplay between gut microbiota, immune signaling, and epigenetic modifications in shaping the glioma microenvironment. This study advances our understanding of the gut-brain axis in glioma biology and proposes the EPHA pathway as a promising biomarker for the immune-mediated modulation of tumor progression, thereby providing new insights into the role of the gut-brain axis in glioma regulation.

## 1 Introduction

The gut microbiota plays a multifaceted role in regulation of host homeostasis, immune function, and metabolic processes, with its influence extending to the modulation of cancer development and progression. Dysbiosis, characterized by an imbalance in the microbial communities, has been linked to various malignancies, including colorectal, liver, breast, and even pancreatic cancers. In colorectal cancer, for example, pathogenic species such as *Fusobacterium nucleatum* have been implicated in tumor promotion by activating pro-inflammatory pathways like NF-κB and promoting immune evasion by modulating tumor-infiltrating myeloid cells (1). Similarly, in hepatocellular carcinoma, gut dysbiosis has been shown to enhance liver inflammation through the translocation of lipopolysaccharides (LPS), which activate Toll-like receptor 4 (TLR4) signaling on hepatic macrophages, fostering a pro-tumorigenic environment (2). Beyond inflammation, gut microbiota-derived metabolites, such as short-chain fatty acids (SCFAs) and secondary bile acids, are known to influence cancer cell metabolism, immune cell differentiation, and even apoptosis. For example, certain bacterial taxa can produce SCFAs like butyrate, which have been shown to exert anti-inflammatory and anti-cancer effects by regulating immune responses and inhibiting histone deacetylases, thus affecting gene expression in cancer cells (3). In addition to its role in tumorigenesis, gut microbiota is emerging as a key modulator of cancer therapies (4). In preclinical models, the administration of *Akkermansia muciniphila* has been demonstrated to restore the efficacy of PD-1 immunotherapy (5). These researches illustrating the diverse mechanisms by which the microbiota regulates cancer progression.

Glioblastoma (GBM), the most aggressive and lethal primary brain tumor, remains a significant therapeutic challenge. Despite advancements in understanding the molecular underpinnings of GBM, the median survival of patients remains dismal, averaging around 15 months (6). Current treatment strategies primarily target the tumor mass but fail to address the diffuse infiltration of GBM cells into surrounding brain tissue, which leads to inevitable recurrence (7). Additionally, the presence of the blood-brain barrier (BBB) complicates drug delivery, limiting the effectiveness of systemically administered therapies (8). While emerging approaches such as immune checkpoint inhibitors and chimeric antigen receptor (CAR) T-cell therapies show promise, their efficacy has been limited in GBM due to the immunosuppressive tumor microenvironment and the tumor’s remarkable ability to evade immune surveillance (7).

Recent research has begun to uncover the complex interactions between the gut microbiota and the central nervous system (CNS) via the gut-brain axis, particularly in the context of gliomas (9). This bidirectional communication network is mediated by microbial metabolites, immune signaling, and neuroimmune pathways, all of which have been shown to influence brain physiology and pathology, including the progression of brain tumor (10). In gliomas, dysbiosis has been shown to exacerbate tumor progression by enhancing inflammatory responses, altering immune cell infiltration into the tumor microenvironment, and modulating local cytokine production (11). Furthermore, the microbiota can influence systemic immune responses that impact the CNS, leading to changes in tumor-associated macrophage populations that either support or suppress tumor growth (12). By integrating multi-omics approaches, we aim to achieve a comprehensive understanding of the potential mechanisms and pathways through which the gut microbiota influences glioma development.

## 2 Results

### 2.1 Antibiotic treatment induces alteration in gene expression in glioma

**Figure 1:**
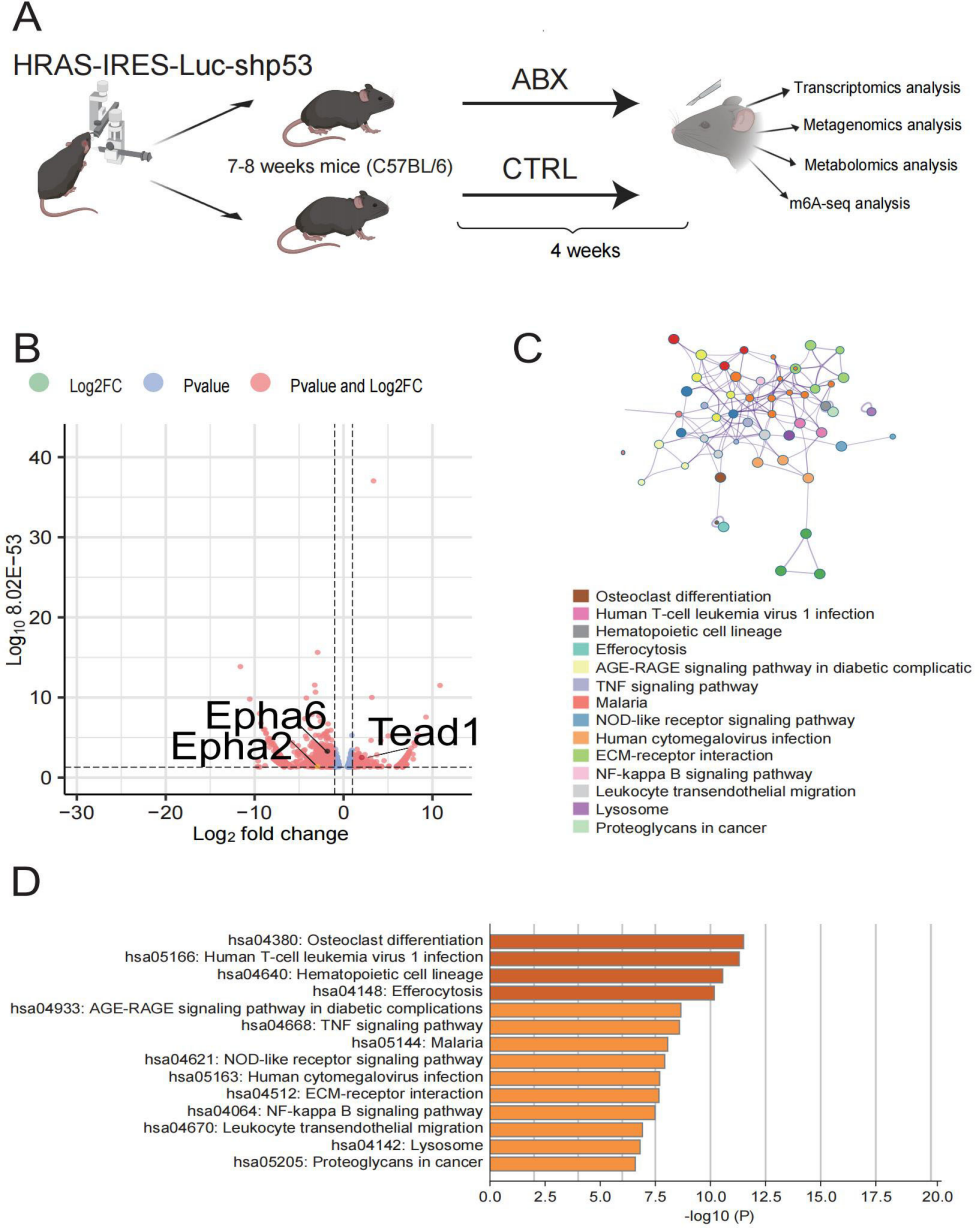
Antibiotic treatment induces the alteration of gene expression in glioma. **(A)** Construction of the GBM mouse model and overall experimental and sequencing workflow. **(B)** Differential expression analysis revealed a significant reduction in the expression of several genes in antibiotic-treated (ABX) mice than those in control (CTRL) group, including Epha6. **(C, D)** Gene Ontology (GO) functional analysis **(C)** and KEGG pathway enrichment analysis **(D)** of the differentially expressed genes in different groups.

We first evaluated the impact of antibiotic treatment on glioma-bearing female mice (**Figure 1A**). Following a four-week ABX treatment, tumor-bearing brains were harvested for downstream analysis. To investigate the molecular mechanisms underlying the observed phenotypic changes, bulk RNA sequencing (RNA-seq) was performed on the tumor samples using the Illumina NovaSeq platform. Differentially expressed genes (DEGs) were identified between the ABX and Control (Ctrl) groups.

Among these DEGs, Epha2, Epha6, and Tead1 exhibited the most significant expression changes (**Figure 1B**). We prioritized these three genes for further investigation based on their critical but distinct roles in oncogenesis: Epha2, a prominent member of the Eph receptor family, has been well-documented to promote the invasion and stemness of glioma stem cells (13, 14); Tead1 serves as a key transcriptional factor in the Hippo pathway, which is heavily implicated in tumor proliferation; while Epha6, despite its poorly characterized role in glioma progression, showed a dramatic response to ABX treatment, making it a compelling candidate for novel regulatory mechanisms.

Consistently, GO enrichment analysis of the DEGs revealed that these altered genes were significantly enriched in malignancy-associated pathways, including the NF-κB signaling pathway, extracellular matrix (ECM)-receptor interaction, and other cancer-related pathways (**Figure 1C**). Individual gene alterations within these cancer-related pathways were further corroborated by barplot analysis (**Figure 1D**).

### 2.2 Single-cell level alteration in ABX-treated GBM samples using ScRNA sequencing analysis

**Figure 2:**
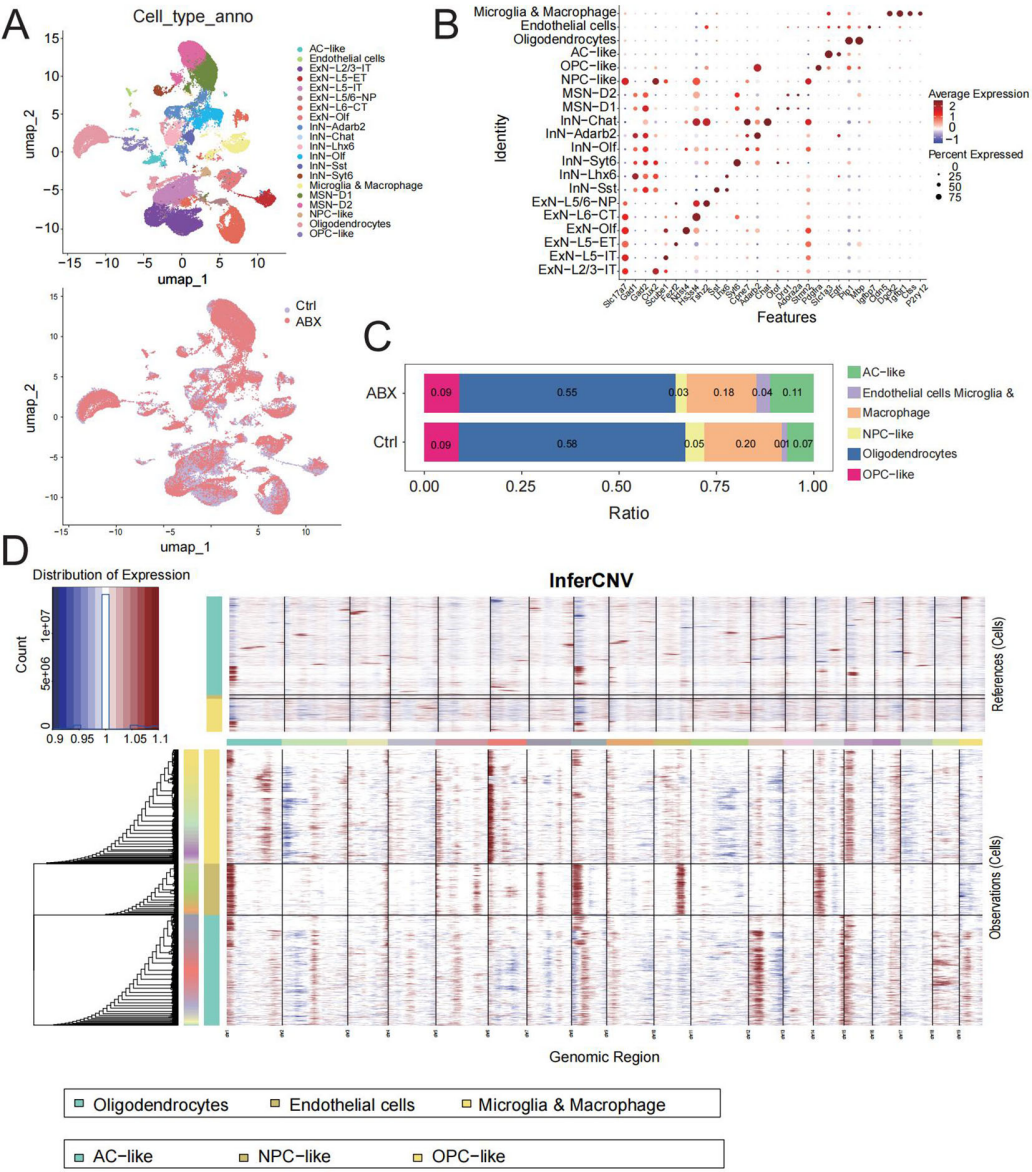
Single-cell level alteration in ABX treated GBM samples using ScRNA sequencing analysis. **(A)** UMAP dimensional reduction plot from single-cell RNA sequencing analysis, showing the spatial distribution of different cell types and groupings in a two-dimensional space. Different colors represent distinct cell types (top) and groups (bottom). **(B)** Dot plot of gene expression levels across different cell types. Dot size represents the proportion of cells expressing the gene, while color intensity reflects the average gene expression level. **(C)** Proportion of key cell types showing significant differences between the two groups. **(D)** InferCNV analysis results. The upper panel displays the gene expression heatmap of reference cells, while the lower panel shows the expression patterns of observed cells. Red indicates gene amplification, blue indicates gene deletion, and the colored stripes represent potential copy number variations (CNVs) across different cell populations.

To further understand the changes in glioma cell types induced by ABX-mediated gut microbiota disruption, we performed single-cell RNA sequencing. UMAP dimensionality reduction revealed distinct spatial distributions of various cell types changed in ABX group GBM mouses, with each color representing a different cell type (**Figure 2A**). We visualized the expression levels and proportions of specific genes within each cell type, where the size of the dots represents the proportion of cells expressing the gene, and the color intensity indicates the average gene expression level (**Figure 2B**). Especially, the proportion of AC-like cell types significantly increased after ABX treatment (**Figure 2C**), suggesting enhanced immune evasion and tumor plasticity (15) and ABX may have specific regulatory effects on AC-like cell type. Additionally, inferCNV analysis identified potential copy number variations (CNVs) in OPC-like, AC-like, and NPC-like cell populations, with red indicating gene amplification and blue indicating gene deletion (**Figure 2D**).

### 2.3 Intercellular signaling networks and gene expression changes

**Figure 3:**
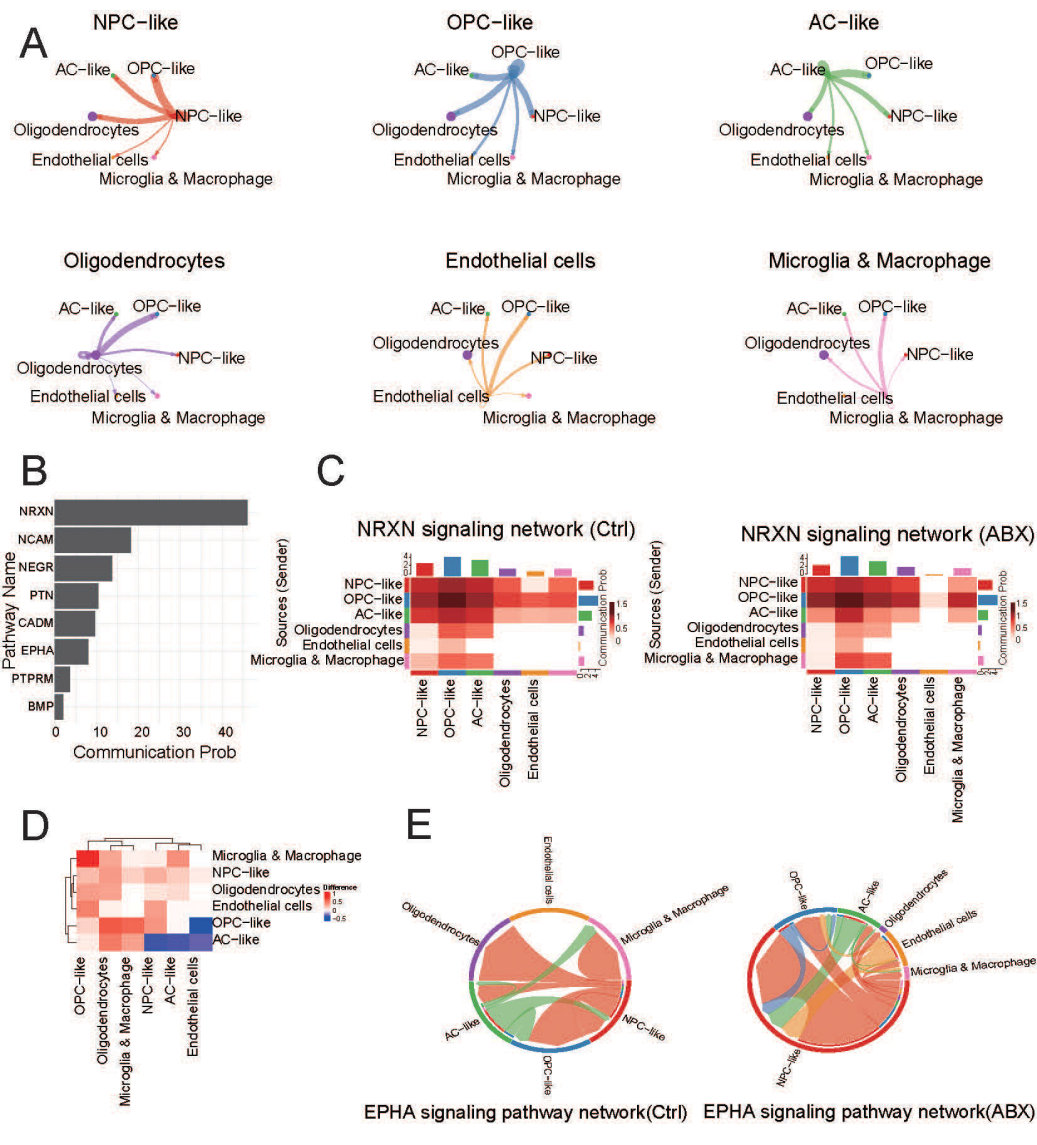
Intercellular signaling networks and gene expression changes in GBM mouse model treated with ABX. **(A)** Intercellular signaling networks between different cell types. Each subpanel illustrates the interaction strength between one cell type and others, with arrow thickness and color indicating the direction and intensity of signaling. **(B)** Ranked list of the most prominent cell communication regulatory networks. **(C)** Visualization of the NRXN signaling pathway across different groups. **(D)** Differential analysis of the NRXN signaling network between pre- and post-ABX treatment, highlighting reduced communication between AC-like, NPC-like, and endothelial cells after ABX treatment. **(E)** Visualization of the EPHA signaling pathway shows significant alterations in cell communication networks between the ABX-treated and control groups.

To further explore the interactions between different cell types, we constructed intercellular signaling networks. It reveals the signaling relationships within various cell types, including NPC-like, OPC-like, AC-like, oligodendrocytes, endothelial cells, and microglia/macrophages (**Figure 3A**). The thickness and color of arrows represent the direction and strength of signaling, with OPC-like cells exhibiting the most extensive communication with other cell types (**Figure 3A**). The NRXN pathway plays a pivotal role in intercellular communication (**Figure 3B**). Further visualization of intercellular signaling highlights the extensive connections between OPC-like and AC-like cells (**Figure 3C**). Using heatmap, we mapped the NRXN signaling pathway across various cell types, underscoring the central roles of OPC-like, AC-like, and NPC-like cells within this network (**Figure 3C**). We then quantified the differences in NRXN signaling between ABX-treated and control groups, revealing a weakened interaction between AC-like cells and both NPC-like and endothelial cells following ABX treatment (**Figure 3D**). This suggests that ABX can influence intercellular communication involving AC-like cells. Interestingly, we also observed significant changes in the expression levels of the glioma-related gene EPHA following ABX treatment. Notably, EPHA-mediated signaling also exhibited marked alterations pre- and post-ABX treatment, indicating that antibiotic-induced changes in EPHA expression likely further regulate intercellular communication (**Figure 3E**).

### 2.4 Spatial distribution of metabolites and gene expression in glioma

**Figure 4:**
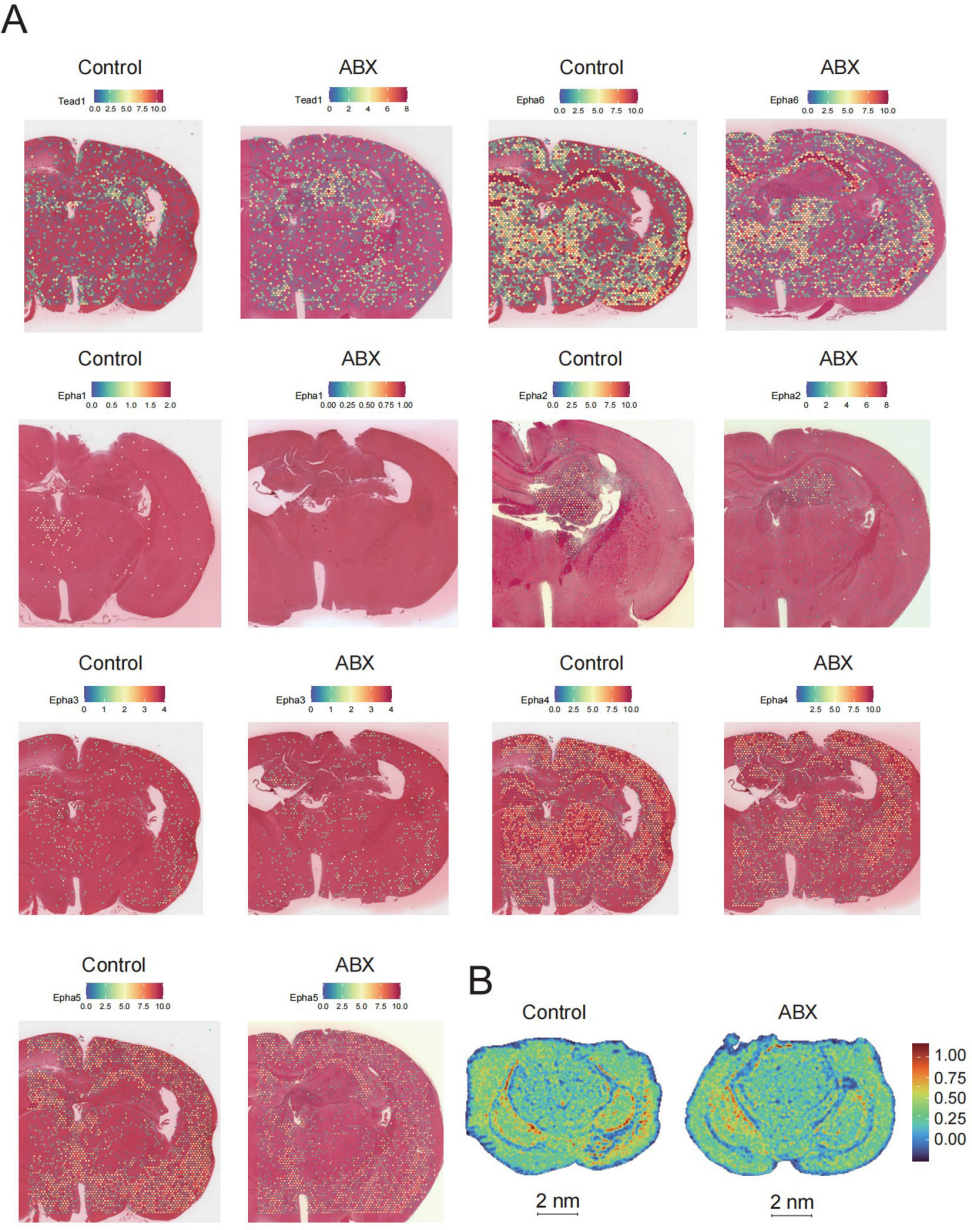
Spatial distribution of metabolites and gene expression in glioma. **(A)** Expression of Tead1 and Epha genes in the brain tissues of control and ABX-treated mice, with different colors representing varying expression levels. The expression level of Tead1 is higher, while expression of Epha6, 1, 2, 3, 4, and 5 under the same experimental conditions were all notably decreased, in the ABX group. **(B)** Distribution of methionine in the brain tissues of control and ABX-treated mice. Redder colors indicate higher metabolite concentrations. Methionine levels are reduced in the ABX-treated group.

We subsequently employed spatial transcriptomics to systematically assess the spatial expression levels of EPHA family genes, such as Epha6, in glioma tissues. Our analysis revealed that key genes in the EPHA pathway—particularly Epha6 and related genes—were significantly downregulated in the GBM regions of the ABX-treated group compared to the control group (**Figure 4A**). In contrast, Tead1 was markedly upregulated in the ABX group (**Figure 4A**). In addition, spatial metabolomic analysis of glioma tissues demonstrated a reduction in methionine distribution in the ABX-treated glioma tissues (**Figure 4B**). These results indicate that ABX treatment may affect the metabolism of intestinal-derived metabolites such as methionine in glioma. Given the critical role of methionine in tumorigenesis (9), this alteration is likely to further promote the malignant phenotype of glioma cells, such as increases in the proportion of AC-like cells previously observed. This suggests the potential role of intestinal-derived metabolites such as methionine in regulating gliomas.

### 2.5 Antibiotic treatment induces changes in the gut microbiome of GBM mice

**Figure 5:**
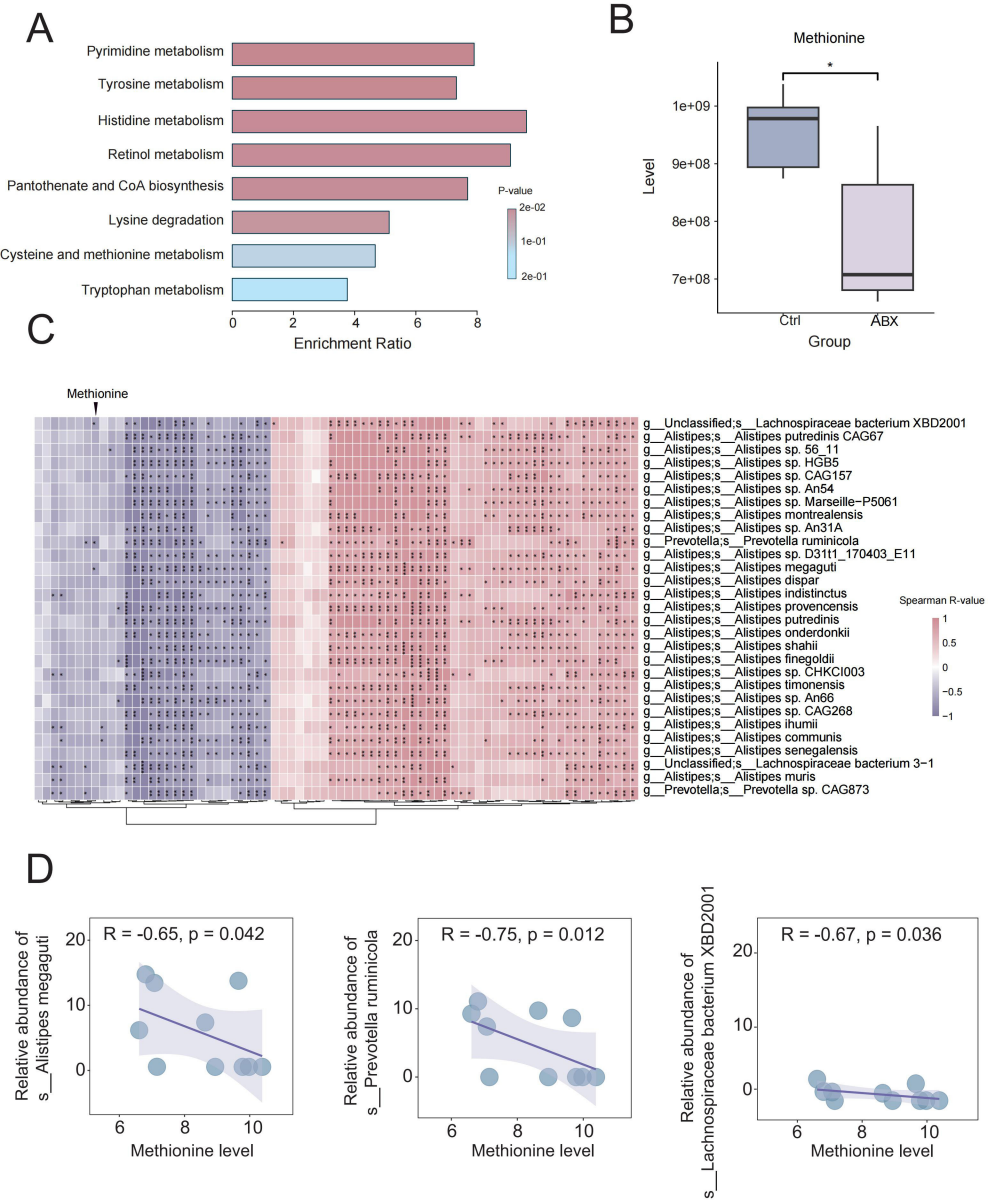
Antibiotic treatment induces changes in the gut microbiome of GBM mice. **(A)** KEGG pathway enrichment of differentially expressed metabolites between ABX and Ctrl groups. **(B)** Student’s t-test analysis of methionine levels between the ABX and Ctrl groups (P < 0.05). **(C)** Heatmap showing the correlation between the relative abundance of methionine-synthesizing microorganisms and several differential metabolites, including methionine (*P < 0.05; **P < 0.01; ***P < 0.001; ****P < 0.0001). **(D)** Correlation between methionine levels and the abundance of methionine-synthesizing microbes (left: s:*Alistipes megaguti*; center: s:*Prevotella ruminicola*; right: s:*Lachnospiraceae* bacterium XBD2001). Spearman correlation was used, with the gray bands indicating the 95% confidence intervals.

The question is how does the gut microbiome influence GBM? To evaluate the gut microbiota in GBM mice, we performed a metagenomic analysis of fecal samples from both the ABX-treated group and Ctrl group. We also conducted a differential analysis of metabolites between the two groups, aiming to identify key differential metabolites. Using MetaboAnalyst, we performed KEGG pathway enrichment analysis of these metabolites. The results revealed that the differential metabolites were primarily enriched in the pyrimidine metabolism, tyrosine metabolism, histidine metabolism, and retinol metabolism pathways (**Figure 5A**).

A Student’s t-test showed significant differences in the levels of methionine between the ABX group and the Ctrl group (**Figure 5B**). Furthermore, we conducted a Spearman correlation analysis to assess the relationship between the abundance of microbiota associated with methionine synthesis (9) and the levels of differential metabolites (**Figure 5C**). This analysis uncovered significant correlations between several differential metabolites, including methionine, and specific microbial taxa (**Figure 5C**). Notably, methionine was significantly correlated with s:*Alistipes megaguti*, s:*Prevotella ruminicola*, and s:*Lachnospiraceae* bacterium XBD2001 (**Figure 5D**).

### 2.6 Distribution of m6A modification peaks and motif analysis in glioma

**Figure 6:**
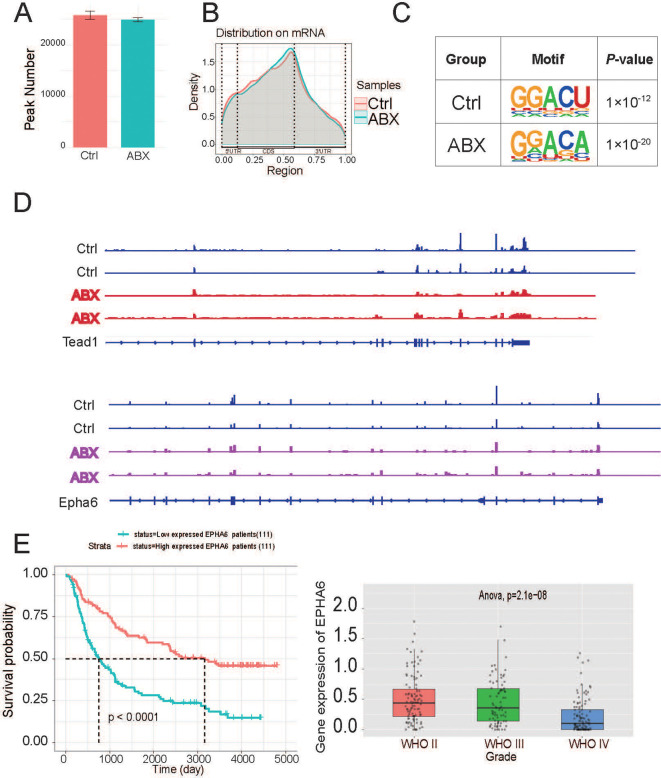
Distribution of m6A modification peaks and motif analysis in glioma. **(A)** Comparison of the number of m6A peaks between the two groups. **(B)** Distribution of m6A peaks across different mRNA regions. **(C)** Motif comparison of the most common m6A sequences between the two groups, indicating highly similar motifs with significant enrichment (P<0.0001). **(D)** Distribution of m6A modification peaks in the Tead1 and Epha6 genes across the two groups shows reduced m6A modifications in both genes following ABX treatment, suggesting that antibiotics may affect m6A modifications of specific genes. **(E)** Prognostic characteristics of EPHA6 in GBM from the CGGA database, along with expression levels of EPHA6 in different stages of brain tumors.

Methionine is known to play a key role in regulating RNA methylation through m6A modification. Therefore, we hypothesized that changes in the gut microbiota, leading to a reduction in methionine levels, may result in decreased m6A methylation, subsequently affecting gene expression. To test this hypothesis, we performed m6A-seq analysis on samples collected before and after ABX treatment. We first compared the number of m6A modification peaks between the control group and the antibiotic-treated (ABX) group, and it revealed a reduction in the number of m6A peaks in the ABX group (**Figure 6A**). Interestingly, compared to the control group, the ABX treatment group showed fewer m6A peaks in the 5’UTR and CDS front end, while displaying more m6A peaks in the CDS back end (**Figure 6B**). This indicates that ABX treatment elicits distinct m6A modification profiles in different regions, pointing to region-dependent regulation. We also examined the most common m6A sequence motifs in both groups and found that the motifs were highly similar, with significant enrichment (p < 0.01); however, the ABX-treated group showed a stronger preference for A-base modifications (**Figure 6C**). Gene-specific analysis identified a reduction in m6A modifications on the Tead1 and Epha6 genes in the ABX group (**Figure 6D**), suggesting that antibiotic treatment may alter the m6A methylation of these genes. Further analysis of Epha6 expression in GBM revealed that its expression was significantly associated with prognosis and tumor stage (**Figure 6E**). In summary, our multi-omics approach provides a comprehensive map of how the gut microbiome influences GBM and uncovers potential molecular mechanisms underlying these interactions.

## 3 Discussion

Our findings provide compelling evidence that gut microbiota dysbiosis, induced by antibiotic treatment, might be involved in GBM progression through immune modulation and epigenetic alterations. The gut-brain axis, a bidirectional communication network between the gut microbiota and central nervous system (CNS), plays a crucial role in regulating these interactions (16–19). Antibiotic-induced dysbiosis resulted in downregulation of EPHA6, a gene previously implicated in tumorigenesis and immune evasion (20), and upregulation of Tead1, known for its involvement in tumor growth and resistance to therapy in glioma (21). EPHA signaling is also linked to tumor angiogenesis, supporting blood supply and tumor growth (22). These alterations in EPHA and Tead1 signaling likely enhance glioma cell migration and invasion, contributing to the tumor’s aggressiveness. These results suggest that dysbiosis might be involved in glioma progression by modulating key signaling pathways and altering tumor microenvironment interactions.

Single-cell transcriptomics revealed that ABX treatment induced a notable shift in glioma cell populations, with a significant increase in AC-like cell types, suggesting a potential role for the microbiota in modulating glioma cell plasticity and immune evasion. The intercellular signaling analysis indicated that the NRXN and EPHA pathways, particularly in AC-like and NPC-like cells, were significantly disrupted following gut microbiota perturbation. These findings support the emerging concept that the gut-brain axis regulates immune and no-immune pathways that contribute to tumor heterogeneity and progression (23).

Recent studies have demonstrated that microbial metabolites can influence host RNA modifications, potentially altering m6A dynamics and, consequently, affecting glioma function and immune responses (24, 25). m6A, the most prevalent internal mRNA modification in eukaryotic cells, plays a crucial role in post-transcriptional regulation, influencing mRNA stability, splicing, translation, and degradation (26–28). Increasing evidence suggests that m6A modifications are dynamically regulated in response to environmental stimuli, including changes in microbial communities (26). Our spatial transcriptomics and metabolomics data revealed a marked reduction in methionine distribution in glioma tissues of the antibiotic-treated mice, a finding that underscores the potential link between gut-derived metabolites and tumor metabolism (29). Methionine, a precursor for S-adenosylmethionine (SAM), is crucial for RNA methylation through m6A modifications. We found that reduced methionine levels were associated with altered m6A methylation patterns, particularly in the 3′ UTR of Tead1 and Epha6, suggesting that gut microbiota disruption may influence glioma progression through epigenetic mechanisms. In the context of the gut-brain axis, m6A-mediated gene regulation may serve as a molecular link through which gut microbiota exert influence on neurodevelopment and neurological disorders (30). The differential regulation of m6A across key pathways, including the NRXN pathway, suggests that microbial dysbiosis could perturb m6A-dependent processes, thereby impacting synaptic function and cognitive outcomes.

Our study opens a new avenue for exploring m6A as a regulatory nexus between the microbiome and the central nervous system, particularly in the context of glioma. However, this study also has some limitations, including potential off-target effects of antibiotic treatment; lack of mechanistic validation; absence of validation using human clinical samples; and limited sample size for multi-omics analyses. Furthermore, incorporating experiments capable of distinguishing between microbiota-dependent effects and the direct effects of antibiotic therapy—such as fecal microbiota transplantation (FMT), germ-free mouse validation studies, and/or microbiota re colonization experiments—would significantly enhance the reliability of the conclusions presented in this study. And further research is needed to elucidate the precise mechanisms through which microbiota-induced changes in m6A influence the gut-brain axis and how these modifications may contribute to disease pathogenesis. Understanding these interactions could pave the way for novel therapeutic strategies targeting m6A regulation in immune and microbiome-related neurodegenerative and neurodevelopmental disorders.

## 4 Materials and methods

### 4.1 Plasmid and lentivirus production

The method for lentivirus production followed protocols established in our previous work. Lentiviral vectors were engineered to carry the HrasG12V oncogene and sh-p53 to generate a GBM model. Specifically, the lentiviral construct for inducing GBM in mice included the HRASV12 fragment and either a sh-p53 fragment. Co-expression of HRASV12 and luciferase was achieved using an Internal Ribosome Entry Site (IRES). To produce the lentivirus, the vector was co-transfected with the packaging plasmids pCMVΔ8.9 and pMD2.G into HEK293T cells. The supernatant containing the lentiviral particles was collected and concentrated by centrifugation at 4 °C, 130, 000 g for 2.5 hours. The resulting viral particles were resuspended in phosphate-buffered saline (PBS) containing 0.1% bovine serum albumin (BSA). Quantification of lentiviral particles was performed using quantitative PCR (qPCR).

### 4.2 Animal models

C57BL/6J mice, aged 6–8 weeks, female, were obtained from GemPharmatech Co., Ltd (Jiangsu, China). Lentivirus was injected stereotactically into the hippocampus using a stereotaxic apparatus (RWD 68001) as described previously (31). A 0.8 mm cranial drill bit was used to thin the skull, followed by injection of 2 µL of purified lentivirus particles. Injection coordinates were 1.6 mm lateral, 1.2 mm anterior to bregma, and 1.75 mm below the skull surface. After injection, the needle was left in place for 3 minutes and then slowly withdrawn over 1 minute. All animal procedures were approved by the Ethics Committee of Guangxi Medical University, and mice were housed under pathogen-free conditions. Mice were treated with antibiotics (ABX: 0.5 g/L vancomycin, 1 g/L penicillin, metronidazole, and neomycin) or vehicle in autoclaved water, with treatment administered continuously for 4 weeks and refreshed every 2 days. The mice were sacrificed by cervical dislocation after being anesthetized with carbon dioxide. All procedures were carried out in accordance with the Guide for the Care and Use of Laboratory Animals, as published by the National Institutes of Health, and adhered to the ARRIVE guidelines.

### 4.3 RNA sequencing analysis

Fresh tissues were processed using the Qiagen RNA Lipid Tissue Extraction Kit, and RNA quality was assessed using TapeStation. Sequencing was performed with paired-end 150 bp reads on the Illumina NovaSeq 6000 platform. Quality control of the sequencing data was conducted using the FastQC package (v0.11.9). The reads were aligned to the mouse reference genome (mm10) using Hisat2 (v2.2.1) with default settings (32). Following alignment, StringTie (v1.3.3b) was used for assembly and quantification, generating count matrices and transcripts per million (TPM) for each gene (33).

### 4.4 m6A sequencing analysis

We conducted m6A sequencing on two groups of GBM tissues (Control and ABX groups with intermittent fasting). Hisat2 (v2.2.1) was used to align the sequences to the mm10 genome, producing BAM files (32). Peak calling and differential peak analysis were performed using the R package exomePeak2 (v1.16.0). For m6A feature analysis, motif enrichment was carried out using HOMER with parameters set to line=1000 and size=200. The distribution of m6A peaks was visualized with the Guitar package (v2.20.2), and coverage tracks were generated in bigWig format using bamCoverage (34, 35). Finally, IGV was employed to visualize the m6A-seq data for both Control and ABX groups.

### 4.5 Single-cell RNA sequencing analysis

We performed single-cell RNA sequencing (scRNA-seq) on GBM tissues from both Control and ABX groups. Sequencing data were aligned and quantified using the CellRanger platform (v5.1.0) from 10X Genomics (35). The resulting gene expression matrix was processed with Seurat (v5.1.0) for quality control and filtering, applying the following criteria: minGene < 500, maxGene < 7000, maxUMI < 50000, pctMT < 1%, pctRB < 1%, ensuring high-quality cells were retained for downstream analysis. After normalization and batch effect correction using Harmony, dimensionality reduction was performed using principal component analysis (PCA) (dims = 1:20, resolution = 0.7) followed by clustering. Data visualization was achieved through uniform manifold approximation and projection (UMAP), and cell types were defined based on marker genes, including tumor cells (NPC-like, OPC-like, AC-like) and immune/supporting cells (Microglia & Macrophages, Oligodendrocytes, Endothelial cells). Feature plots were used to display the proportion and average expression levels of characteristic genes across cell types. To assess genomic instability in different cell populations, we used inferCNV (v1.20.0) to infer copy number variations (CNVs) (36). Oligodendrocytes and endothelial cells served as reference populations, and CNV states in OPC-like, AC-like, and NPC-like populations were inferred. To elucidate intercellular interactions, we utilized CellChat (v1.6.1) to construct cell signaling networks, calculating the likelihood of each cell type acting as a signal sender, receiver, or mediator based on normalized gene expression data (36).

### 4.6 Spatial transcriptomics analysis

Tissues excised from GBM mice were formalin-fixed and embedded in paraffin. After ensuring RNA quality control (DV200 > 30%), 5-micron sections were mounted onto Visium gene expression slides. RNA was released by reversing crosslinking with 0.1N HCl and TE buffer (pH 9.0), followed by overnight hybridization with three specific probes (5’ end containing small RNA Read 2S and 3’ end containing poly-A). cDNA libraries were constructed according to the 10X Genomics protocol and sequenced on the NovaSeq 6000 platform (Illumina). Post-sequencing, data alignment and gene quantification were conducted using Space Ranger (v2.0.1) from 10X Genomics. The conversion of FASTQ files was handled via the spaceranger mkfastq pipeline, followed by the spaceranger count pipeline to align reads to the mm10 mouse reference genome, calculate unique molecular identifiers (UMIs), and generate a matrix of spatial feature points corresponding to the histological image. Data analysis was performed in R using Seurat (v5.1.0) with the Load10x_Spatial command to analyze spatial transcriptomics data (37).

### 4.7 Metabolomics analysis

Five biological replicates were performed for each group, and blood samples were used for metabolomic profiling. After homogenization and incubation on ice, the samples were centrifuged and injected into an LC-MS/MS system for analysis (38). Elution was performed using an optimized gradient analysis strategy to ensure comprehensive metabolite coverage, with the mass spectrometer operating in both positive and negative ion modes. Peak alignment, extraction, and quantification were carried out using Compound Discoverer 3.3. Data were normalized to total ion current, and molecular formulas were predicted. Peaks were matched to databases such as mzCloud and mzVault for qualitative and quantitative results. KEGG, HMDB, and LIPIDMaps were used for metabolite annotation. Principal component analysis (PCA) and partial least squares discriminant analysis (PLS-DA) were conducted with metaX. Univariate analysis (t-test) was used to compute statistical significance (P-value). Metabolites with a VIP > 1, P-value < 0.05, and |log2 FC| > 1 were considered differentially expressed (39). For clustering heatmaps, data were standardized by Z-scores of the differential metabolite intensities, and heatmaps were plotted using the pheatmap R package (v1.0.12) (37). KEGG enrichment analysis was conducted using the online tool MetaboAnalyst 6.0 (https://www.metaboanalyst.ca/).

### 4.8 Spatial metabolomics analysis

Tissues were cryosectioned at a thickness of 10 µm, and the sections were transferred onto pre-cooled ITO slides. After tissue transparency, they were dried by rubbing. 2, 5-Dihydroxybenzoic acid (DHB) matrix was sprayed onto the tissue sections using a TM-Sprayer. The ITO slides were mounted onto a mass spectrometry target plate, and the tissue regions were identified using DataImaging software. Laser scanning was employed to induce ionization, and the released molecules were analyzed to obtain mass-to-charge ratios (m/z) and peak intensity data. Raw data were processed using SCiLS Lab software for smoothing and root mean square (RMS) normalization, generating relative intensity data for m/z ratios. The mass spectrum was fine-tuned using 3D point cloud data. Detected metabolites were qualitatively and quantitatively analyzed using MSI mode, and their spatial metabolic distribution was visualized in 3D images.

## Data Availability

All data are made freely available in GSA database (https://bigd.big.ac.cn/gsub/) and OMIX database (https://ngdc.cncb.ac.cn/omix/), including RNA-seq (CRA015912, https://ngdc.cncb.ac.cn/gsa/s/L7941E7i), m6A-seq (CRA015927, https://ngdc.cncb.ac.cn/gsa/s/L7941E7i)(CRA019762, https://ngdc.cncb.ac.cn/gsa/s/76rG1Gd6), scRNA-seq (CRA016372, https://ngdc.cncb.ac.cn/gsa/s/fe9G0g6L)(OMIX007725, https://ngdc.cncb.ac.cn/omix/preview/oSVIQE8T), spatial transcriptomics data (OMIX006329, https://ngdc.cncb.ac.cn/omix/preview/3NMQPfH9)(OMIX007655, https://ngdc.cncb.ac.cn/omix/preview/6SfaaK2a), metabolomics data (OMIX006398, https://ngdc.cncb.ac.cn/omix/preview/l9JmoR0Z), and metagenomic data (CRA015939, https://ngdc.cncb.ac.cn/gsa/s/HHjvy63Q).
